# Suppression of the Savoyard University Schools

**Published:** 1861-01

**Authors:** 


SUPPRESSION OF THE SAVOYARD UNIVERSITY SCHOOLS.—The *Medical Times and Gazette*, November 10th, 1860, states that a decree, published in the *Moniteur*, suppresses the University schools of Theology, Law, Medicine, and Pharmacy, established at Chambéry, Nice, Annecy, St. Jean-de-Maurienne, Moutiers, Bonneville, and Thonon, declaring that diplomas of Doctors in Medicine and Pharmacy, obtained at Sardinian Universities prior to January, 1861, by persons native of Sardinia—and henceforth by annexation Frenchmen—will be considered as equivalent to French diplomas.

